# Diverse bacterial consortia: key drivers of rhizosoil fertility modulating microbiome functions, plant physiology, nutrition, and soybean grain yield

**DOI:** 10.1186/s40793-024-00595-0

**Published:** 2024-07-19

**Authors:** Luiz Gustavo Moretti, Carlos Alexandre Costa Crusciol, Marcio Fernandes Alves Leite, Letusa Momesso, João William Bossolani, Ohana Yonara Assis Costa, Mariangela Hungria, Eiko Eurya Kuramae

**Affiliations:** 1 College of Agricultural Sciences, Department of Crop Science, São Paulo State University (UNESP), Botucatu, São Paulo, 18610-034 Brazil; 2 Department of Microbial Ecology, Netherlands Institute of Ecology (NIOO-KNAW), Wageningen, 6708 PB The Netherlands; 3 Embrapa Soybean, Carlos João Strass Highway, Post Office Box 231, Londrina, Paraná 86001-970 Brazil; 4 Institute of Environmental Biology, Ecology and Biodiversity, Utrecht University, Padualaan 8, Utrecht, 3584 CH The Netherlands; 5 School of Agriculture, Federal University of Goiás (UFG), 74690-900, Goiânia, Goiás Brazil

**Keywords:** Bacterial consortium, *Glycine max*, Rhizomicrobial ecology, Shotgun metagenomics, Plant growth-promoting bacteria

## Abstract

Soybean cultivation in tropical regions relies on symbioses with nitrogen-fixing *Bradyrhizobium* and plant growth-promoting bacteria (PGPBs), reducing environmental impacts of N fertilizers and pesticides. We evaluate the effects of soybean inoculation with different bacterial consortia combined with PGPBs or microbial secondary metabolites (MSMs) on rhizosoil chemistry, plant physiology, plant nutrition, grain yield, and rhizosphere microbial functions under field conditions over three growing seasons with four treatments: standard inoculation of *Bradyrhizobium japonicum* and *Bradyrhizobium diazoefficiens* consortium (SI); SI plus foliar spraying with *Bacillus subtilis* (SI + Bs); SI plus foliar spraying with *Azospirillum brasilense* (SI + Az); and SI plus seed application of MSMs enriched in lipo-chitooligosaccharides extracted from *B. diazoefficiens* and *Rhizobium tropici* (SI + MSM). Rhizosphere microbial composition, diversity, and function was assessed by metagenomics. The relationships between rhizosoil chemistry, plant nutrition, grain yield, and the abundance of microbial taxa and functions were determined by generalized joint attribute modeling. The bacterial consortia had the most significant impact on rhizosphere soil fertility, which in turn affected the bacterial community, plant physiology, nutrient availability, and production. Cluster analysis identified microbial groups and functions correlated with shifts in rhizosoil chemistry and plant nutrition. Bacterial consortia positively modulated specific genera and functional pathways involved in biosynthesis of plant secondary metabolites, amino acids, lipopolysaccharides, photosynthesis, bacterial secretion systems, and sulfur metabolism. The effects of the bacterial consortia on the soybean holobiont, particularly the rhizomicrobiome and rhizosoil fertility, highlight the importance of selecting appropriate consortia for desired outcomes. These findings have implications for microbial-based agricultural practices that enhance crop productivity, quality, and sustainability.

**Graphical Abstract:**

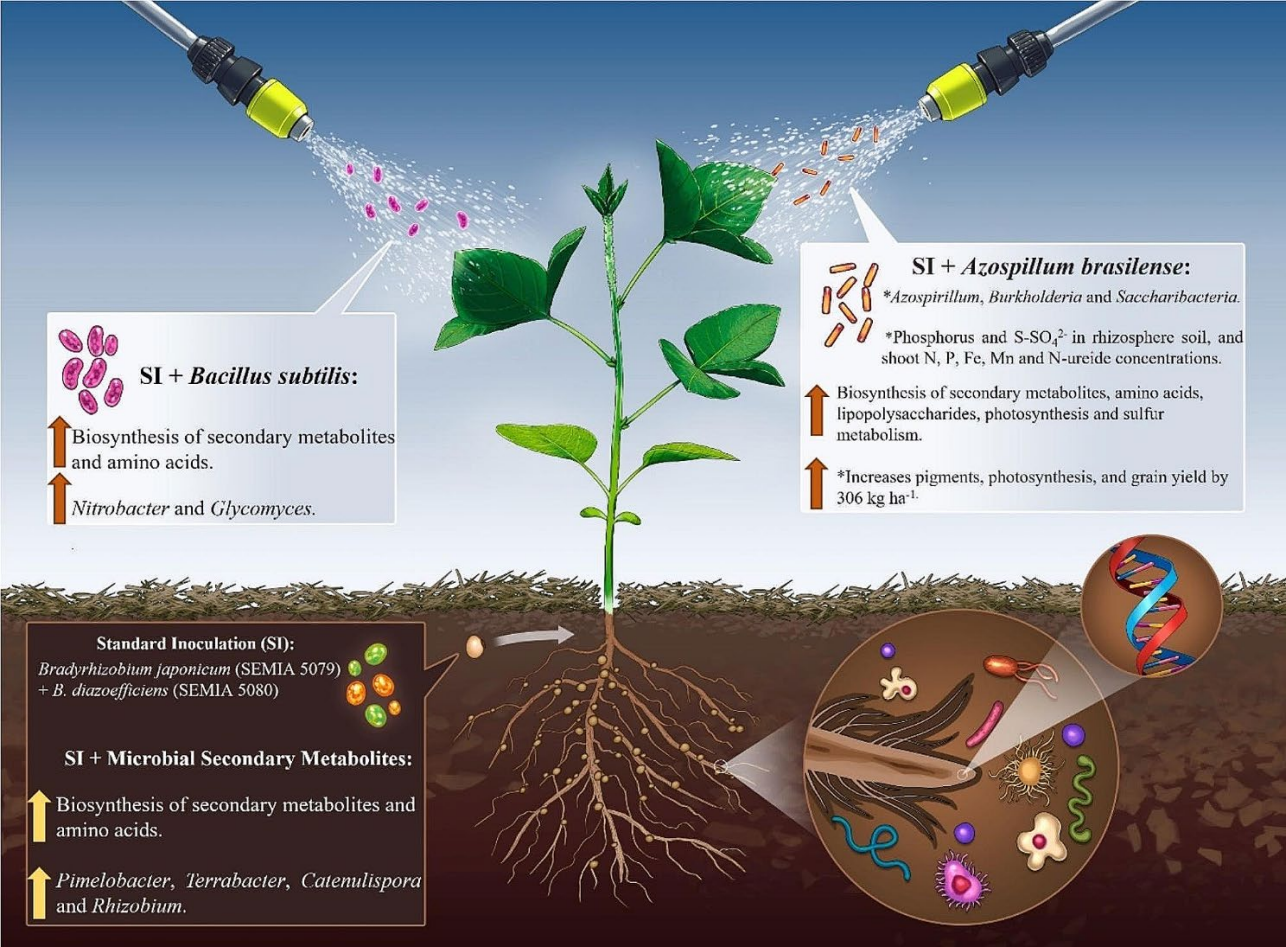

**Supplementary Information:**

The online version contains supplementary material available at 10.1186/s40793-024-00595-0.

## Introduction

The rhizosphere microbiome impacts plant growth, development and health, and provides essential functions for nutrient acquisition, abiotic and biotic stress tolerance, protection against soil-borne pathogens, and host immune regulation. The rhizosphere is the soil immediately surrounding and influenced by the roots of a plant [1–3]. Roots influence the soil both physically via root structure or heat generation and chemically by secreting plant metabolites.

Soybean (*Glycine max* L. Merrill) is a major crop worldwide, with global production of more than 340 million tons annually [4]. The success of soybean cultivation under tropical conditions depends in part on the symbiosis of the plant with *Bradyrhizobium* and biological nitrogen fixation (BNF), which provides nitrogen (N) and ensures high yields without supplemental mineral N application [5, 6]. This symbiosis has implications for agricultural sustainability efforts, which seek to reduce the use of agricultural inputs and the environmental impacts of agriculture through economic, social and environmental approaches [7].

*Bradyrhizobium*, *Azospirillum*, *Bacillus* bacteria, and lipo-chitooligosaccharides play a crucial role in soybean cultivation in tropical conditions [8]. *Bradyrhizobium* enhances nitrogen fixation, directly supplying essential nutrients to the plants [9]. *Azospirillum* boosts root development and improves stress resistance [10]. *Bacillus* species contribute to disease suppression and soil health improvement [11]. Lipo-chitooligosaccharides act as signaling molecules, promoting symbiotic interactions and enhancing nutrient uptake [12]. Together, these elements support sustainable and productive soybean farming in tropical regions.

It is challenging to understand and quantify the impacts of plant growth-promoting bacteria (PGPB) on the rhizosphere and the whole plant. The diverse organic nutrients (e.g., organic acids, phytosiderophores, sugars, vitamins, amino acids, nucleosides, and mucilage) and signals exuded by plant roots attract microbes that can thrive in this habitat [13, 14]. The microbial community associated with plant roots is referred rhizomicrobiome. It promotes plant development by promoting resistance to soil-borne pathogens [15], attenuating abiotic stresses such as salinity, drought and aluminum [16, 17], increasing nutrient uptake [18], and promoting plant growth and nutritional quality [19, 20]. However, mechanisms underlying the interactions of soybean inoculated with consortium of PGPB with various microbes in the rhizosphere remain especially elusive. Hence, here we address the questions: (*i*) How does inoculation with different combinations of PGPBs and microbial metabolites influence soybean plant development, yield and grain quality, and rhizosphere soil fertility? (*ii*) How do the taxonomic and functional diversities of the microbial community assemblage of the rhizosphere microbial communities change when soybean is inoculated with different PGPBs and their metabolites? To answer these questions, we compared the impacts of inoculation with nitrogen-fixing *Bradyrhizobium* spp., plant growth-promoting *Azospirillum brasilense*, the biocontrol agent *Bacillus subtilis*, and/or rhizobial metabolites on crop nutrition, photosynthetic pigments, grain yield, and rhizosphere soil chemical analysis, and shotgun metagenomics approach. This experimental design aims to elucidate the synergistic effects of various beneficial microbes and their metabolites on soybean rhizosphere microbiome, providing insights into sustainable and productive farming practices in tropical regions.

## Materials and methods

### Site description and experimental design

Field experiments were carried out at Lageado Experimental Farm of São Paulo State University, Botucatu, São Paulo State, Brazil. Three experiments were conducted during the 2016–2017, 2017–2018 and 2018–2019 growing seasons. The geographical, climate and soil attributes are summarized in Table 1 and Figure S1.

**Table 1:** Geographic coordinates, climate, soil physicochemical attributes, and crop management of the experimental area. Botucatu, Brazil

Site Description	Value	Unit
**Geographical coordinates**		
Latitude	22°83′3′′ S	^**°**^
Longitude	48°42′6′′ W	^**°**^
Sea level	765	m
**Climate attributes**		
Climate classification^a^	Mesothermal climate	Cwa
Annual precipitation ^b^	~ 1,360	mm
Air temperature (minimum)	15.3	^°^C
Air temperature (maximum)	26.1	^°^C
**Soil physical attributes** ^c^		
Soil type^d^	Typic Haplorthox	–
Clay	602	g kg^− 1^
Silt	281	g kg^− 1^
Sand	117	g kg^− 1^
Bulk density	1.19	g cm^− 3^
**Soil chemical attributes** ^f^		
pH (CaCl_2_)	5.1	–
Soil organic C (SOC)	12.2	g kg^− 1^
Phosphorus–available (P _resin_)	32	mg kg^− 1^
Calcium (Ca^2+^_resin_)	25.0	mmol_c_ kg^− 1^
Magnesium (Mg^2+^_resin_)	15.0	mmol_c_ kg^− 1^
Potassium (K^+^ resin)	3.9	mmol_c_ kg^− 1^
S-Sulfate (S–SO_4_^2−^_Ca(H2PO4)2_)	4.9	mmol_c_ kg^− 1^
Aluminum (Al^3+^_KCl_)	2.0	mmol_c_ kg^− 1^
Total acidity (H + Al) _(at pH 7.0)_	42.0	mmol_c_ kg^− 1^
Base saturation (BS)	51.0	%
Cation exchange capacity _(CEC pH 7.0)_	86.0	%
**Soil biological attributes** ^g^		
Most probable number	9.32 × 10^4^	CFU g^− 1^

^a^ [75]. ^b^ [76]. ^c^ [77]. ^d^ [78]. ^f^Prior to the establishment of the study (2016), the initial soil properties were determined at a depth of 0–20 cm [79]. ^g^ [80]

A randomized complete block design was adopted with four inoculation treatments and four replicates in three growing seasons. The four inoculation treatments were:

(i) SI: Standard inoculation, seed inoculation with *Bradyrhizobium japonicum* (strain SEMIA 5079) + *Bradyrhizobium diazoefficiens* (strain SEMIA 5080) at sowing;
(ii) SI + MSM: SI plus application of microbial metabolites (MSM) extracted from *B. diazoefficiens* (strain USDA 110) + *Rhizobium tropici* (strain CIAT 889) at sowing;
(iii) SI + Bs: SI at sowing plus foliar spraying with *Bacillus subtilis* (Bs) (strain QST 713) at soybean phenological stage V_3_ (Fehr and Caviness, 1977);
(iv) SI + Az: SI at sowing plus foliar spraying with *Azospirillum brasilense* (Ab) (strains Ab-V5 and Ab-V6) at soybean phenological stage V_4_.

Foliar spraying with *(A) brasilense* or *(B) subtilis* performed in the late afternoon (5:00 p.m. local time) was carried out according to the manufacturer’s recommendations. The timing of inoculation was determined in previous studies [18, 21, 22].

### Microbial inoculants and secondary metabolites

Liquid inoculants containing 7 × 10^9^ colony-forming units (CFUs) mL^− 1^ of *B. japonicum* strain SEMIA 5079 (= CPAC 15, =CNPSo 07) and *B. diazoefficiens* strain SEMIA 5080 (= CPAC 7, =CNPSo 06) were applied to provide 1.2 × 10^6^ cells seed^− 1^. The Brazilian program for selecting natural variants of *B. japonicum* and *B. diazoefficiens* adapted to environmental conditions [23], and the strains used in this study are representative of the majority of inoculants applied in tropical soybean cultivation.

Lipo-chitooligosaccharide (LCO)-enriched MSMs were extracted from *R. tropici* strain CIAT 899 (= CNPSo 103, = SEMIA 4077) and *B. diazoefficiens* strain USDA 110 (= CNPSo 56). Bacterial cultivation and extraction of the supernatant with *n*-butanol were performed as previously described [24]. MSMs were purified by solid-phase chromatography on a Resprep C18 solid-phase extraction (SPE) column (Teknokroma Analitica S.A., Barcelona, Spain), concentrated and lyophilized. Prior to soybean sowing, the lyophilized MSMs were resuspended in 20% acetonitrile in water, and the concentration was adjusted to 1.0 mL L^− 1^ (approximately 10^− 8^ M). Two hundred milliliters of suspension were applied per 50 kg of seeds [22].

For foliar spraying with *B. subtilis* strain QST 713 at soybean phenological stage V_3_ [25], 3 L of inoculant containing 1 × 10^9^ CFU mL^− 1^ was diluted in 200 L of water per ha. For foliar spraying with *A. brasilense* strain Ab-V5 (= CNPSo 2083) and strain Ab-V6 (= CNPSo 2084) at soybean phenological stage V_4_ [25], 300 mL of inoculant containing 1.2 × 10^5^ CFU mL^− 1^ was diluted in 150 L of water per ha.

The*B. japonicum*, *B. diazoefficiens* and *A. brasilense* strains have been deposited in the “Diazotrophic and Plant Growth Promoting Bacteria Culture Collection of Embrapa Soja” (WFCC Collection # 1213, WDCM Collection # 1054) in Londrina, State of Parana, Brazil. *Bacillus subtilis* strain QST 713 was developed by Bayer CropScience (European Union, Reg. (EC) no. 839/2008).

### Crop management practices history

In each of the three growing seasons, soybean cultivar TMG 1264 RR (Tropical Breeding & Genetics^®^, Paraná, Brazil) was sown after black oat (*Avena strigosa* Schreb.), which had been cropped in the winter as a mulch crop under no-till system and provided an average of 5 Mg ha^− 1^ straw in a dry land area (without irrigation). In a given plot, the specified treatment was applied in each of the three growing seasons. The field plots consisted of ten rows with a length of 10 m and a spacing of 0.45 m, leading to a total plot area of 45 m^2^. The plots were separated by 0.5-m-wide rows and 1.5-m-wide terraces to avoid cross-contamination from surface runoff containing bacteria or fertilizers due to heavy rainfall. In each growing season and plot, fertilization with 00 − 20 − 20 N-P_2_O_5_-K_2_O was performed at 300 kg ha^− 1^. For inoculation, the seeds were evenly coated with the appropriate amount of inoculant 1 h before sowing. Foliar spraying was performed using a tractor-mounted sprayer. The management of weeds, insects and diseases was carried out according to the recommendations [26] when necessary to ensure that these were not limiting factors.

### Crop nutrition

Plant nutritional status was evaluated at soybean phenological stage R_2_ (full bloom) [25] by collecting 20 leaves from the third node from the top in each plot [27]. The plant material was used to determine the concentrations of nitrogen (N), phosphorus (P), potassium (K), calcium (Ca), magnesium (Mg) sulfur (S), copper (Cu), iron (Fe), zinc (Zn), manganese (Mn), and boron (B) [28].

### Photosynthetic pigments

The concentrations of chlorophyll *a*, chlorophyll *b*, total chlorophyll and carotenoids were quantified in fresh diagnostic leaves collected at the same time as the leaves for the nutritional analysis in all growing seasons. The plant tissue was stored in N-dimethylformamide (DMF) and analyzed spectrophotometrically [29].

### Gas exchange parameters

Gas exchange measurements were performed using a model CIRAS-3 portable gas exchange device (PP Systems Inc., Amesbury, MA, USA). The following parameters were measured using diagnostic leaves from plants that had not been collected for: net photosynthetic rate (*A*, µmol CO_2_ m^− 2^s^− 1^); stomatal conductance (*gs*, mol H_2_O m^− 2^s^− 1^); internal CO_2_ (*Ci*, µmol mol^− 1^); transpiration (*E*, mmol H_2_O m^− 2^s^− 1^); water use efficiency (WUE, µmol CO_2_ (mmol H_2_O)^−1^) calculated by the *A/E* ratio and obtained through instant reading; and carboxylation efficiency (Cef) obtained by the *A/Ci* ratio. The system was calibrated with the following parameters: 380–400 ppm atmospheric CO_2_, 1100 µmol quanta m^− 2^s^− 1^ photosynthetically active radiation (PAR) supplied by LED lamps, 25–27 °C leaf chamber temperature, and 60–70% relative humidity. Measurements were carried out under natural conditions on clear days between 10:00 a.m. and 12:00 p.m. with 10 replicates.

### Agronomic parameters and grain yield

In all growing seasons, plants at physiological maturity were harvested from a 16-m^2^ area in the central part of the plot. Grain yield (kg ha^− 1^) and hundred-grain weight (W100G) were converted to dry weight values by correcting for 13% moisture. Moisture content was determined using an automatic moisture meter (model G650i, Gehaka^®^, Goiás, Brazil).

### *Rhizosphere soil sampling*

At the R_2_ flowering stage [25] in December 2018 (the third growing season of the experiment), soybean rhizosphere soil samples were collected from each treatment according to specific technical recommendations [30]. Briefly, the soil loosely attached to the soybean roots was detached by gentle shaking and collected. In addition, three bulk soil samples (0–10 cm) were collected from the standard inoculation plots.

### Rhizosphere soil chemical analysis

Soil samples were processed according to the standard methods for Brazilian tropical soils [31]. All samples were pooled, dried, sieved (2 mm), and analyzed for pH, SOM, P, K^+^, Ca^2+^, Mg^2+^, S–SO_4_^2−^, Fe, Cu, Zn, Mn and B.

### Rhizosphere DNA extraction, shotgun metagenomic sequencing and data processing

Total DNA was extracted from 250 mg of rhizosphere soil sample using the DNeasy PowerSoil Kit (Qiagen, Hilden, Germany) according to the manufacturer’s protocol. The DNA quality and concentration were assessed using a NanoDrop 1000 spectrophotometer (Thermo Scientific, Wilmington, DE, USA) and by 1% sodium boric acid agarose gel electrophoresis [32].

Five libraries (corresponding to bulk soil and the four rhizosphere soil samples) were prepared using NextSeq 500/550 Mid Output v2 (300 cycles; Illumina, San Diego, CA, USA) according to the manufacturer’s protocol for shotgun metagenomic sequencing on the Illumina NextSeq 500 platform (two × 150 bp paired-end). Quality filtering of the reads was performed [33] to remove low-quality sequences (phred score < 20) and short reads ≤ 50 bp using an identity level of 60% and an e-value cut-off of *E* < 1 × 10^− 5^. The sequences were submitted to the European Nucleotide Archive (ENA) and are available under accession number PRJEB31659 (Table S1).

The sequences were processed using the EBI MGnify pipeline [34] and the ATLAS (Automatic Tool for Local Assembly Structures) v2.1.0 pipeline [35] in R software v. 3.1.2 [36]. The EBI MGnify pipeline was used to perform a quality check using Trimmomatic [37], identify RNA gene sequences using Infernal [38] and classify SSU RNA gene sequences against the SILVA 132 database [39] using MAPseq [40]. The ATLAS pipeline was used to check sequence quality using BBDuk [41] and perform cross-assembly (forward and reverse) with Megahit [42]. Functional and taxonomic classifications were assigned at the ORF level for the assembled contigs.

Prodigal [43] was used for ORF prediction, and the eggNOG database [44] was used for functional annotation (COG and KO numbers) using DIAMOND software [45]. EggNOG-mapper [46] was employed for functional annotation of CAZymes with the dbCAN v7 database [47]. Custom scripts were used to generate tables containing information on the taxonomic and functional abundances of the ORFs in all samples.

### Statistical analysis

The agronomic assessments dataset were preliminary analyzed to check for normality of errors using the Shapiro-Wilk test [48] and for homoscedasticity of variances using Levene’s test [49]. Subsequent data analysis was performed in R software [36]. The data were subjected to analysis of variance (ANOVA) with the application of the F test at a 10% probability level. The means were compared using the modified t-test (Least Significant Difference - LSD) with a significance level of 𝑝 ≤ 0.10.

For integrating the data, we used the generalized joint attribute model (GJAM) from the *gjam* R package [50]. GJAM is a hierarchical Bayesian model that belongs to the class of joint species distribution model [51]. It allows to analyze various data types simultaneously by including a censoring approach to the data that is not continuous (e.g., presence-absence, ordinal, discrete, composition) while account for the overdispersion nature of community data [51, 52]. In our study, we used GJAM model to integrate the variables of microbiome (taxa and functions), plant biomass, plant nutrient content, plant physiology, and rhizosphere soil chemical analysis (rhizosoil). As a joint species model that integrates other data types, GJAM offers the possibility to analyze how the different microbes will respond to the treatments and also the other plant and soil variables which are represented in the scale where the data is measured (e.g., relative abundance for microbiome data, grams for plant biomass) in the form of regression coefficients. In our study, given the complexity of our experimental design, we separated the data integration into two models.

In *model I*, we investigated the effect of rhizosphere selection by comparing the differences in microbial taxa and functions between the bulk soil and rhizosphere soil samples from the standard inoculation (SI).

Next, in *model II*, we focused on rhizosphere selection to explore the impact of the different bacterial consortia and metabolites on the microbial community and functions [53]. We extracted regression coefficients from each of the models to identify shifts in the microbial community (taxa and functions) and additional environmental and plant factors. In addition, we performed sensitivity analysis to determine the relative impacts of each treatment on different groups of dependent variables: archaeal, bacterial and fungal communities, rhizosphere soil fertility, plant nutrition, plant physiology and plant production. To check the effects of the treatments in the sensitivity values for the different groups, we performed a multiple comparison Tukey HSD test (*stats* package in R). The models were evaluated using the Markov Chain Monte Carlo (MCMC) method to check if the estimated coefficients reached a stable value (after 6,000 simulations).

The regression coefficients were visualized via cluster trees based on the Ward’s method using Euclidean distances. The same coefficients were used in Principal Component Analysis (PCA) plot to explore the community similarities between treatments and highlight shifts induced by the different treatments. The relevance of the different microbes in the soil community was evaluated by not only the intensities of the shifts in the relative abundances of the soil microbiome provided by the GJAM regression coefficients but also the centered log-ratio (CLR)-transformed abundance [54]. CLR transformation provides information on the relevance of different microbial groups (in our case, taxa and functions) as a proportion of the sample average. We used this transformation to classify the soil microbes as originally high abundance or low abundance based on the log-fold differences in relation to the average, which in the CLR-transformed data corresponds to a value of zero.

## Results

### Plant nutrition

Shoot N, P, Fe, Mn and N-ureide concentrations were 7.1%, 14.3%, 12.8%, 17.7% and 16.7% higher, respectively, in SI + Az than in SI (Table S3). By contrast, K, Ca, Mg, S, Cu, Zn, and B concentrations did not differ between the treatments. In all treatments, nutrient concentrations were adequate for soybean [26].

### Photosynthetic pigments

The concentrations of photosynthetic pigments showed no significant differences between SI + Bs and SI (Table S4), but were higher in SI + Az compared to SI. The highest pigment concentrations were observed in SI + MSM. Specifically, compared to SI, concentrations of chlorophyll *a*, chlorophyll *b*, and total chlorophyll were 12.6%, 26.7%, and 15.0% higher in SI + Az, and 10.0%, 26.7%, and 15.0% higher in SI + MSM, respectively.

### Gas exchange parameters

Photosynthetic activity was higher in SI + MSM and SI + Az than in SI, with increases in net photosynthesis (*A*) of 12.6% and 15.4%, respectively (Table S5). Stomatal conductance (*gs*) and the internal CO_2_ concentration (*Ci*) were 52.5% and 6.3% higher in SI + Az than in SI, resulting in an improvement in water use efficiency (WUE) of 14.9%.

### Plant production

Compared with SI, biomass and nodule parameters were higher in SI + Az (Table S6), with increases of up to 20% in nodule number (NN), 14.8% in nodule dry weight (NDW), 9.6% in root dry weight (RDW), and 3.9% in shoot dry weight (SDW). Significant effects of the treatments on the final population, number of branches per plant, position of insertion of the first pod, and number of grains per pod were not observed (data not shown). In addition, the 100-grain weight (100-GW) was 1.8% higher in SI + Az than in SI, and the grain yield (GY) increased by 306 kg ha^− 1^. As a result, the agronomic efficiency index (AEI) was 6.4% higher in SI + Az than in SI.

### Rhizosphere soil chemical analysis

Table S2 compares the means of 17 rhizosphere soil chemical attributes. The available P, S-SO_4_^2−^, Cu, Fe and Mn concentrations were 17.8%, 12.0%, 10.5%, 8.3% and 25% higher, respectively, in SI + Az than in SI (*p* < 0.001). However, the other rhizosphere soil chemical attributes were not influenced by the treatments.

### Rhizosphere selection

**Fig. 1:**
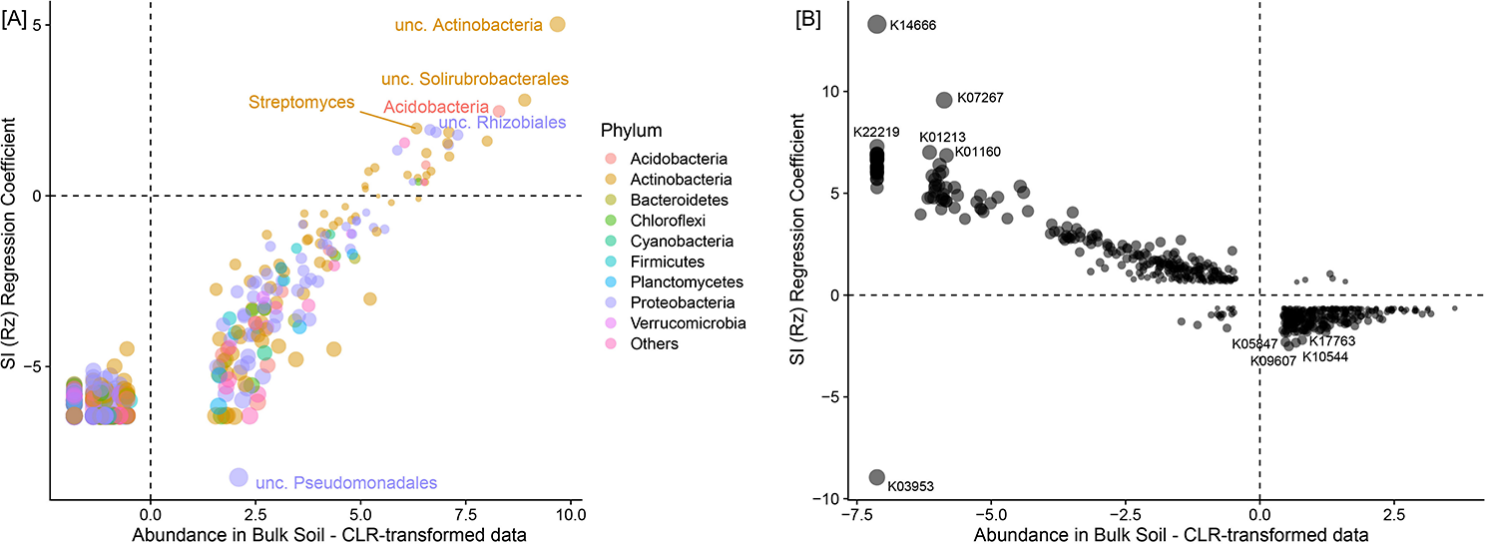
Rhizosphere selection of both (**A**) taxa and (**B**) functions in the standard inoculation (SI) treatment

To visualize the differences in community structure and function between bulk soil and soybean rhizosphere soil in the standard inoculation (SI) (Fig. 1), CLR-transformed data were used. We observed rhizosphere selection of microbial community structure members and their potential functionalities compared with bulk soil; that is, the structure of the soil microbial community in the rhizosphere differed from that in bulk soil. Compared to bulk soil, the rhizosphere generally had higher microbial abundance but lower biological diversity. This is because it harbors microorganisms that are specifically recruited and adapted to their unique environmental conditions.

**Fig. 2:**
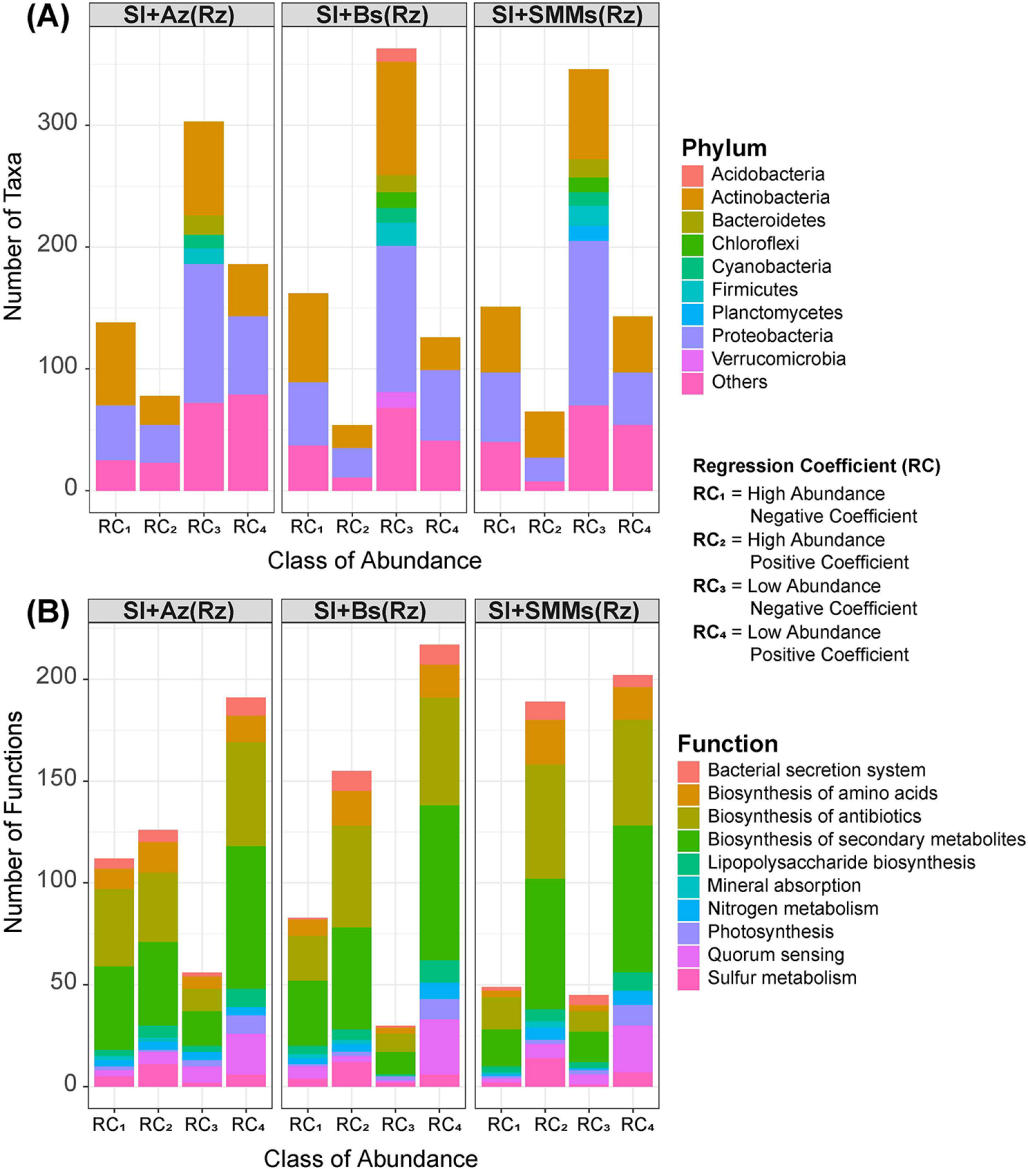
(**A**) Impacts of the four treatments on classes of taxa abundance. (**B**) Impacts of the four treatments on classes of functional abundance

As shown in Fig. 2A, the application of bacterial consortia dramatically reduced the numbers of both high-abundance and low-abundance taxa compared with SI alone. In SI + Az, there were 139 and 76 high-abundance taxa with negative and positive coefficients, respectively, and 301 and 185 low-abundance taxa with negative and positive coefficients, respectively. These values were 162, 51, 362, and 126 in SI + Bs and 150, 63, 344, and 145 in SI + MSM, respectively.

Interestingly, treatment-specific patterns of increases in the numbers of both high- and low-abundance functions were observed (Fig. 2B). The numbers of negative and positive high-abundance functions and negative and positive low-abundance functions were 112, 127, 57, and 192 in SI + Az; 83, 155, 30, and 217 in SI + Bs; and 49, 189, 45 and 200 in SI + MSM, respectively.

### Impacts of the bacterial consortia on the soybean rhizomicrobiome

**Fig. 3:**
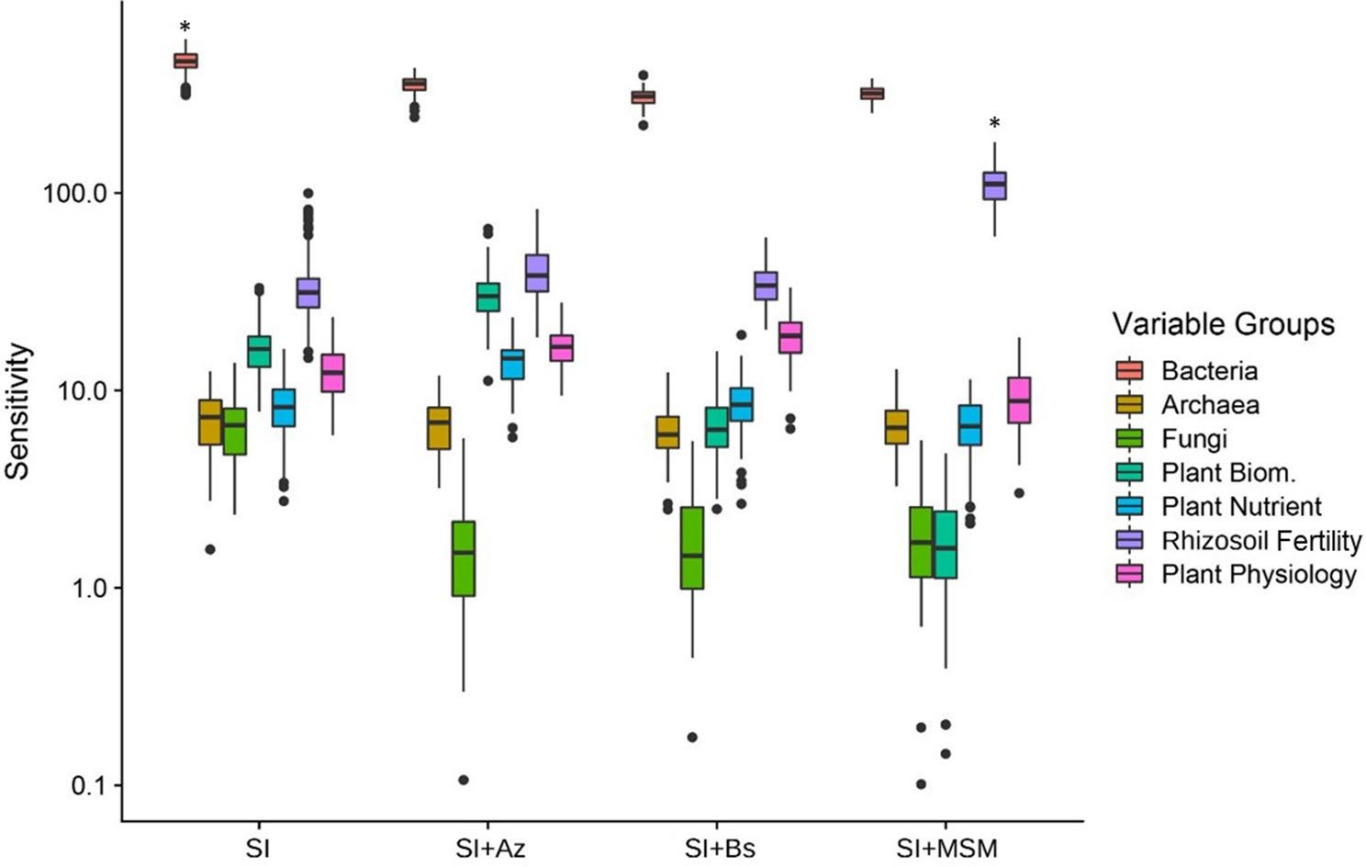
Sensitivity analysis of the holobiont profile according to the four treatments. Treatments with a asterisk (*) are significantly different from the other as suggested by the Tukey HSD post hoc test at 5% probability-level

Sensitivity analysis revealed how the different treatments affected our groups of dependent variables: archaeal, bacterial and fungal communities, rhizosoil fertility, plant nutrition, plant physiology and plant production (Fig. 3). In general, the impact of the treatments was greatest for bacterial community, followed by the rhizosoil fertility, plant physiology, nutrition and production, and the archaeal and fungal communities. In addition, among all treatments, the sensitivity values for the bacterial community were highest for SI and the sensitivity values for the rhizosoil were highest in SI + MSM treatment (*p* < 0.05, Tukey HSD posthoc test).

**Fig. 4:**
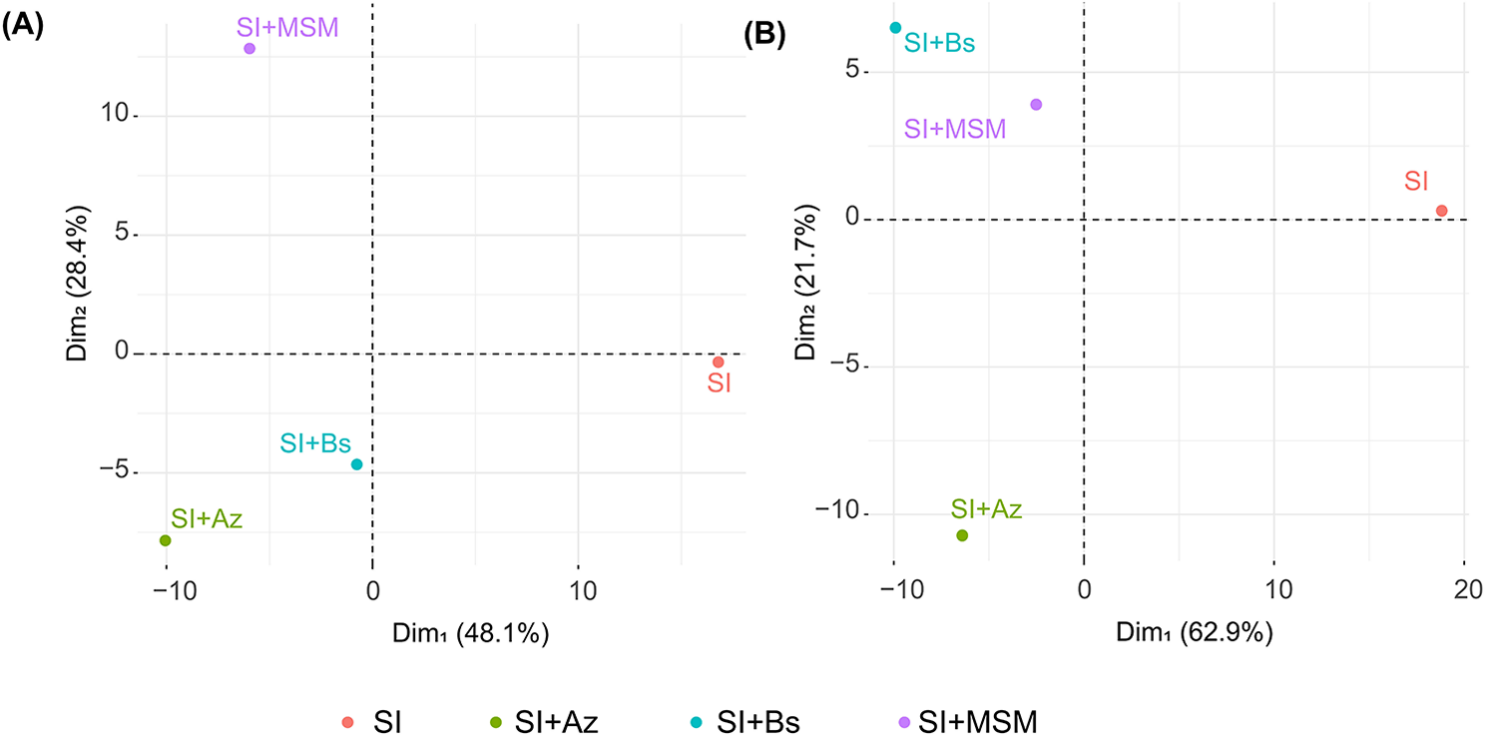
(**A**) Dissimilarity in the holobiont microbial community profile (microbial community structure, plant productivity, plant nutrients, plant physiology, and rhizosoil factors). (**B**) Dissimilarity in the holobiont functional profile (microbial functions, plant productivity, plant nutrients, plant physiology, and soil factors)

Our PCA revealed similarities in the way our treatments influenced the rhizosoil physicochemical parameters and the microbiome (Fig. 4). We observed a clear segregation of the treatments by the first two axes, which explained 76.5% of the total variation in the holobiont microbial community profile (Fig. 4A) and 84.6% of the total variation in the holobiont functional profile (Fig. 4B).

In terms of distance from the control (SI), some of the bacterial consortium treatments displayed high proximity. SI + Az and SI + Bs exhibited high similarity in the sensitivity analysis and PCA of the holobiont microbial community profile (Figs. 3 and 4A), whereas SI + MSM and SI + Bs exhibited high similarity in the PCA of the holobiont functional profile (Fig. 4B), most likely because they produced similar effects on the functional groups.

### Soybean holobiont

**Fig. 5:**
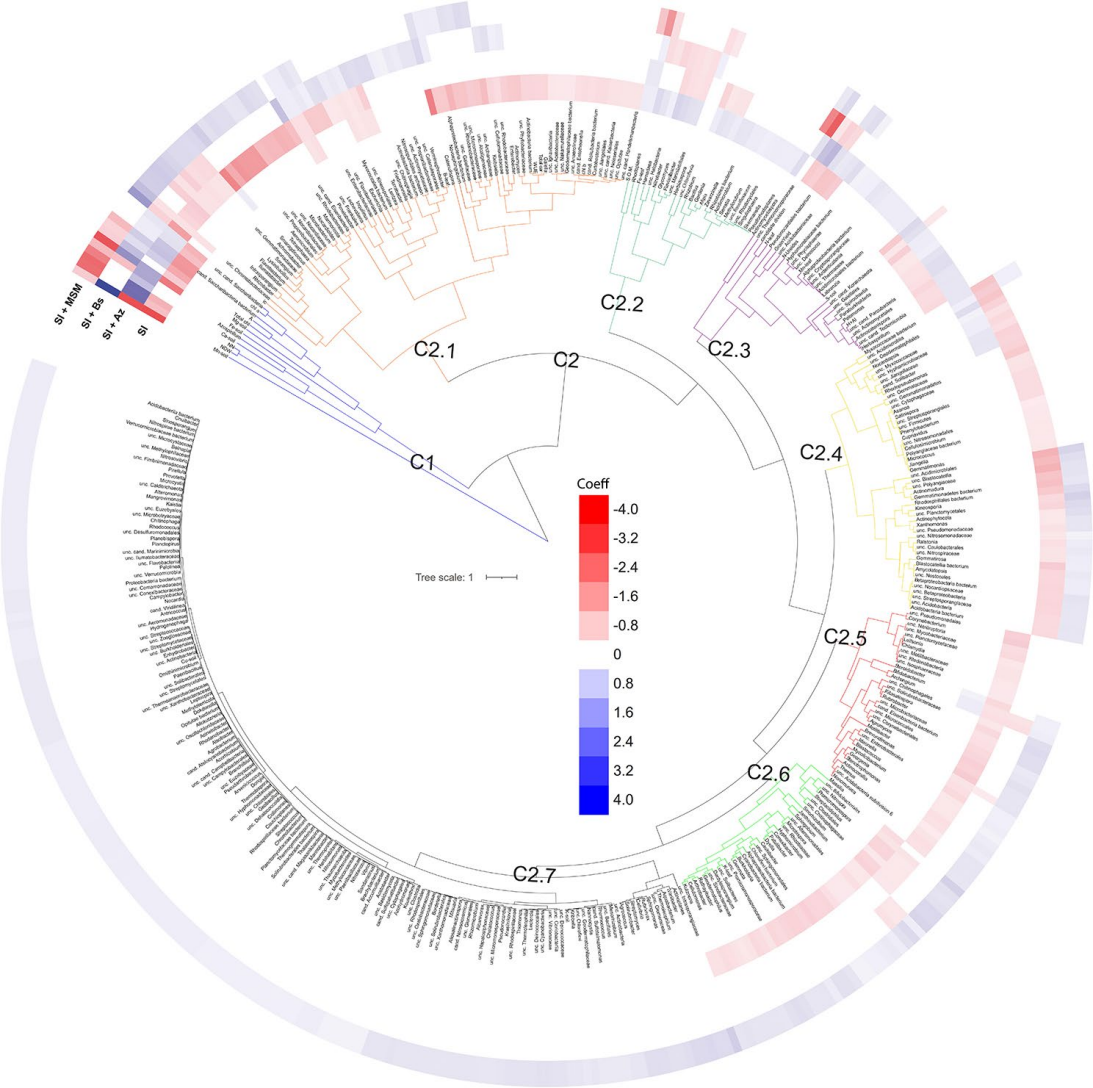
Influence of the bacterial consortia and metabolites on the soybean holobiont (microbial community structure, plant productivity, plant nutrients, plant physiology, and rhizosoil factors)

**Fig. 6:**
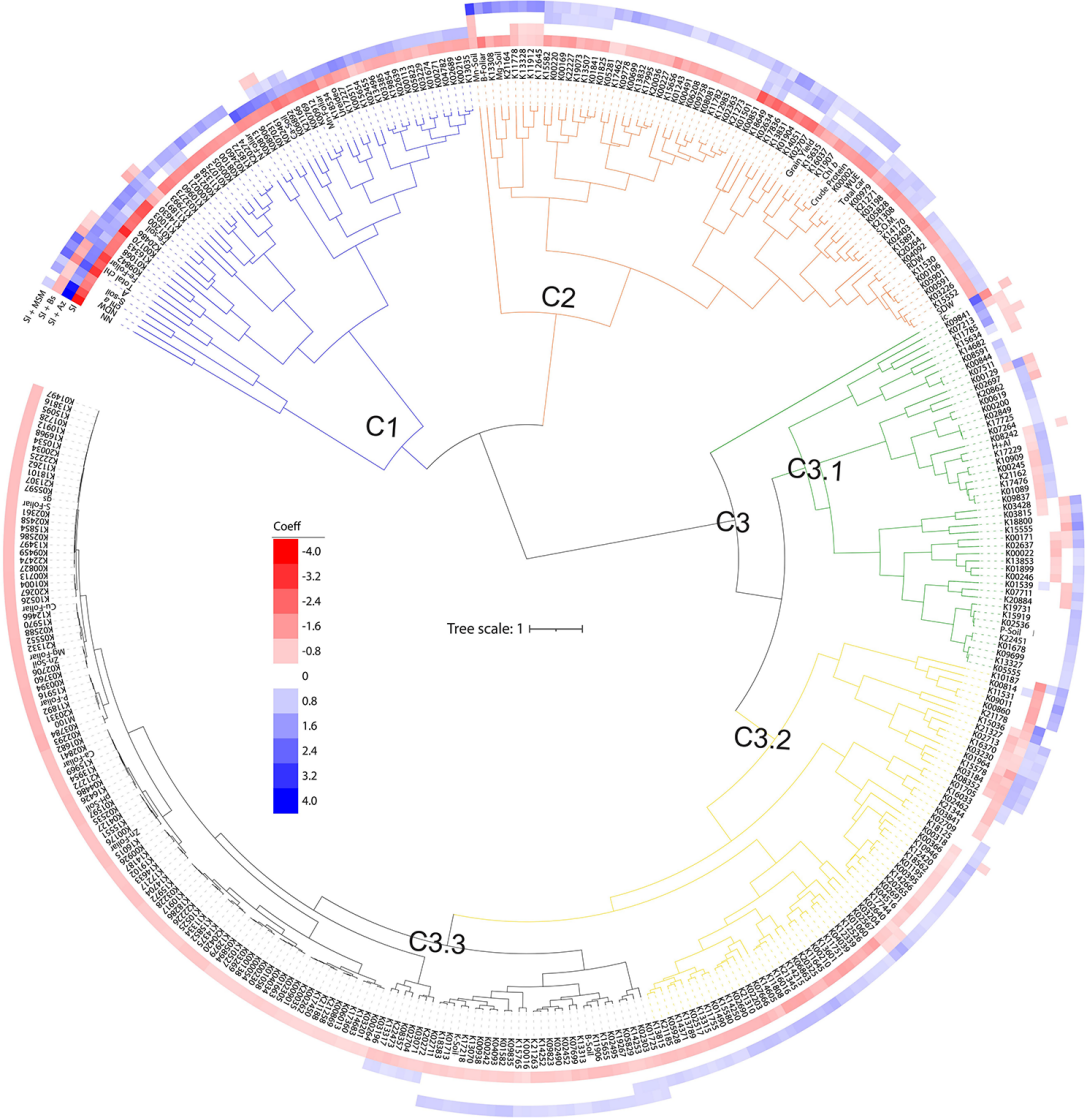
Influence of the bacterial consortia and metabolites on the soybean holobiont (microbial functional structure, plant productivity, plant nutrients, plant physiology, and rhizosoil factors)

Cluster analysis based on regression coefficients identified groups of variables that responded similarly to the treatments. This approach allowed us to discern how chnages in plant and rhizosphere soil physicochemical properties influenced both microbial community structure (Fig. 5) and potential functions (Fig. 6). First, in C1, SI + Az and SI + Bs strongly positively affected rhizosoil fertility (Ca^2+^, Mg^2+^, Fe and Mn), plant attributes (physiology, i.e., chl *a*, total chl and *A*; and biometrics, i.e., NN and NDW), and 3 microbial genera (notably *Azospirillum* spp.) (Fig. 5). In C2.1, SI + Bs and SI + MSM positively influenced plant nutrition (B), plant physiology (chl *b*, total carotenoids, *Ci* and WUE) and 68 microbial genera. In C2.2, SI positively affected rhizosoil fertility (SOM), plant nutrition (Fe) and 23 microbial genera. In C2.3, SI + Az positively impacted rhizosoil fertility (H + Al and S–SO_4_^2−^), plant nutrition (N and Mn), plant physiology (N-ureides) and 24 microbial genera. By contrast, in C2.4, SI + Bs negatively affected 49 microbial genera, and in C2.5, SI + Az and SI + MSM negatively and positively affected 33 genera, respectively. Finally, in C2.6, 32 genera and foliar K were negatively influenced by SI but positively impacted by SI + MSM, and in C2.7, SI + MSM strongly positively influenced rhizosoil fertility (K^+^ and Cu) and 152 genera.

Cluster analysis of functions (Fig. 6) revealed that in C1, SI + Az strongly positively affected most plant attributes (nutrition, i.e., N, Fe and Mn; physiology, i.e., chl *a*, total chl, *A* and N-ureides; and biometrics, i.e., NN and NDW), rhizosoil fertility (Ca^2+^, S–SO_4_^2−^ and Fe) and 47 potential functional pathways, suggesting positive associations between the microbes in these clusters and these plant and soil properties. In C2, SI was inferior to the other treatments mainly in plant attributes (nutrition, i.e., B; physiology, i.e., chl *b*, total carotenoids, and WUE; biometrics, i.e., RDW, SDW, yield and grain crude protein), rhizosoil fertility (SOM, Mg^2+^ and Mn) and 61 potential functional pathways. C3 segregated into distinct clusters (C3.1-3). In C3.1, the bacterial consortia strongly positively affected plant physiology (*Ci*), rhizosoil fertility (H + Al and P) and 45 potential functional pathways. In C3.2, SI + Bs strongly positively affected 67 potential functional pathways. Finally, in C3.3, SI strongly negatively impacted plant nutrition (P, Ca^2+^, Mg^2+^, S–SO_4_^2−^, Zn and Cu), rhizosoil fertility (pH, K^+^, B and Zn) and 124 potential functional pathways but positively influenced plant physiology (*gs*) and biometrics (W100G).

### Shifts in the rhizomicrobiome

**Fig. 7:**
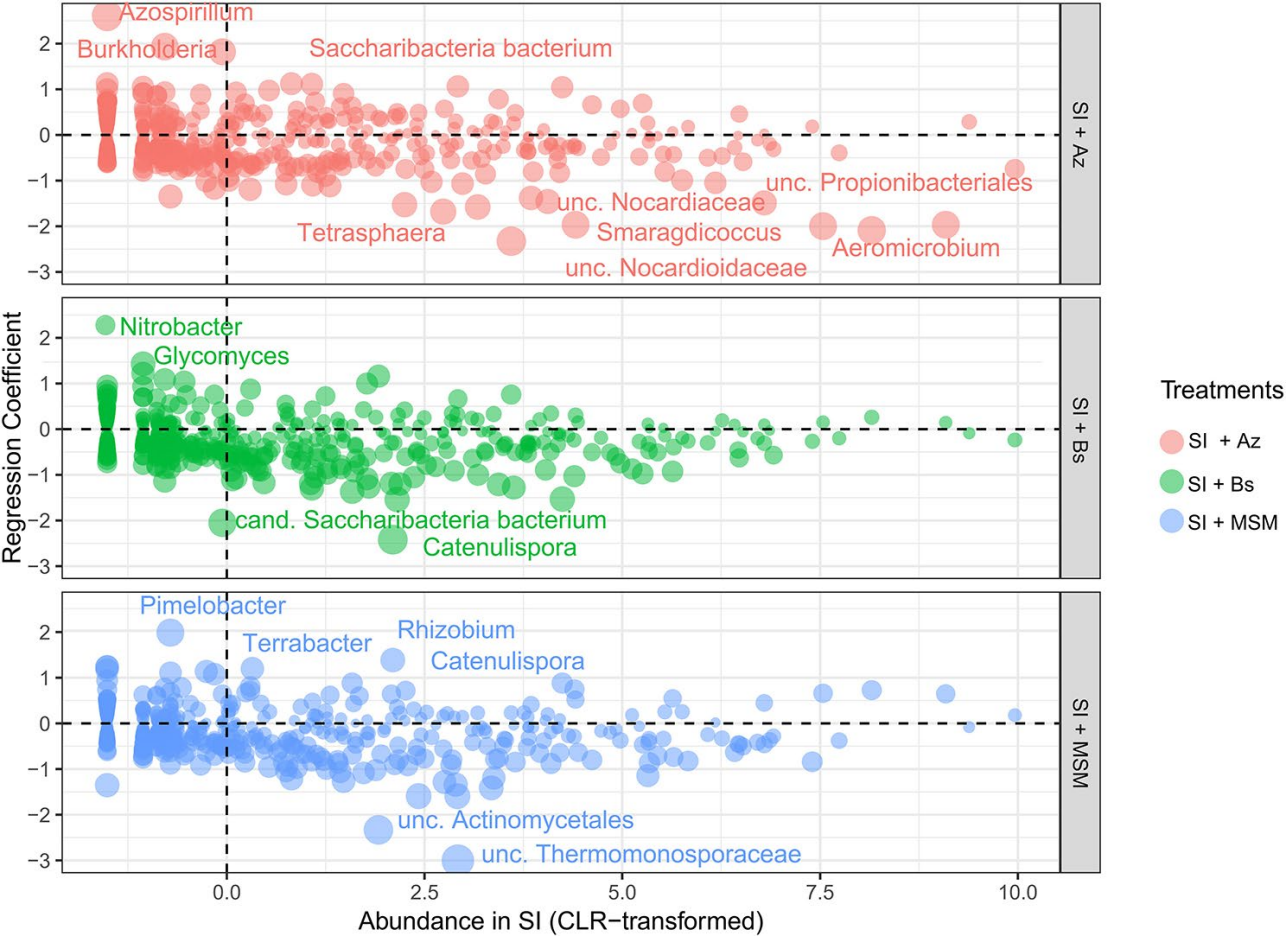
Shifts in the soil microbiome induced by the bacterial consortia or metabolites compared with the standard inoculation

**Fig. 8:**
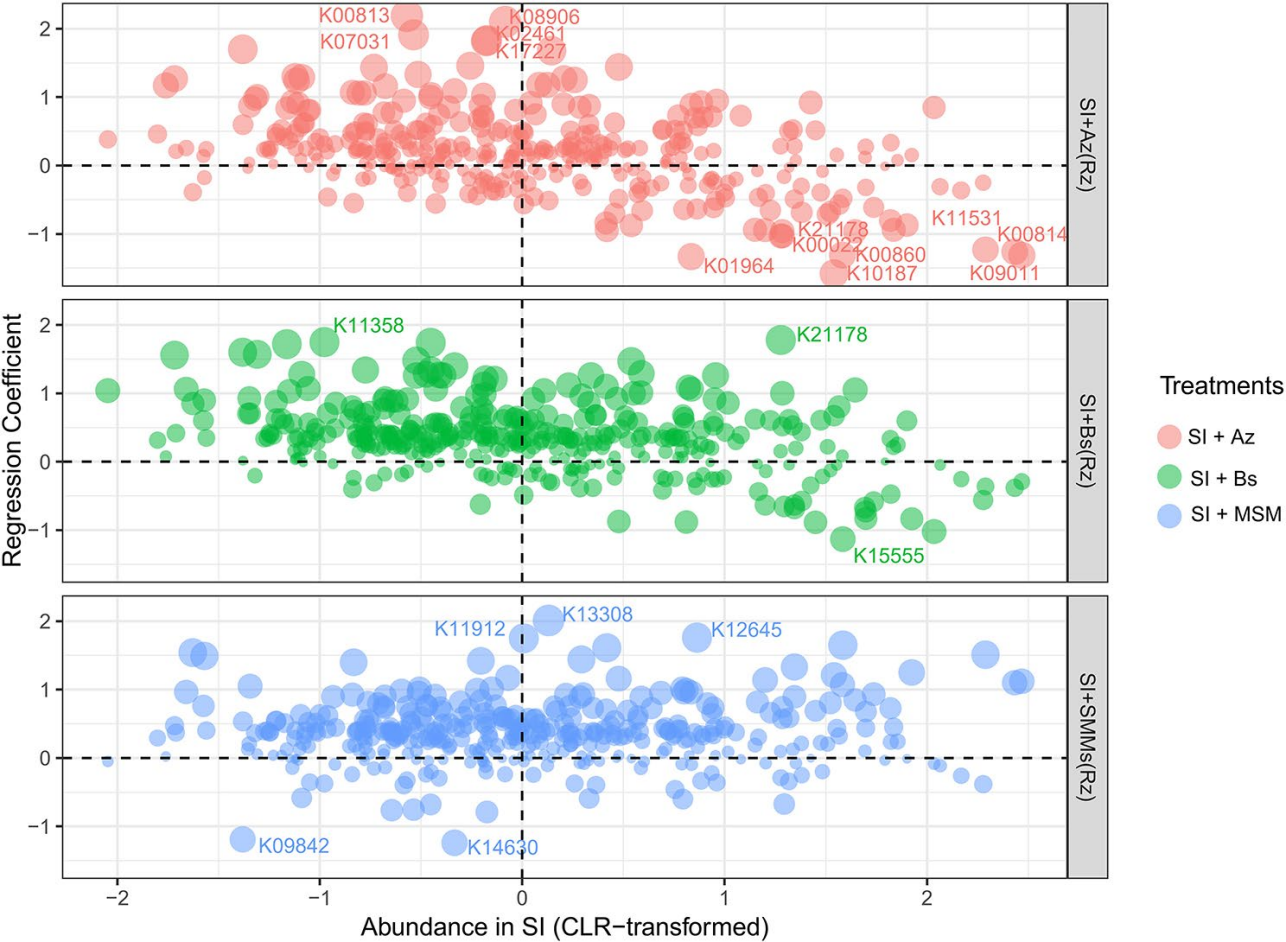
Shifts in the potential functional profile induced by the bacterial consortia or metabolites compared with the standard inoculation

Compared with SI, SI + Az primarily positively modulated *Azospirillum*, *Burkholderia* and *Candidatus Saccharibacteria* and the following functions: K00813 (aspC) (Figs. 7 and 8), which is part of 14 pathways linked to the biosynthesis of secondary metabolites and amino acids; K07031 (hddA), a component of 3 pathways linked to lipopolysaccharide biosynthesis; K08906 (petJ), which belongs to 2 pathways linked to photosynthesis; K02461 (gspL), which is part of 2 pathways linked to bacterial secretion systems; and K17227 (soxZ), a member of 3 pathways linked to sulfur metabolism.

SI + Bs mainly affected *Nitrobacter* and *Glycomyces* simultaneously with the following functions: K21178 (sgcD4), which is part of 3 pathways linked to the biosynthesis of secondary metabolites, and K11358 (yhdR), a component of 14 pathways linked to the biosynthesis of secondary metabolites and amino acids (Figs. 7 and 8). Finally, SI + MSM positively influenced *Pimelobacter*, *Terrabacter*, *Catenulispora* and *Rhizobium* concurrently with the following functions: K11912 (ppkA), which is part of two pathways linked to bacterial secretion systems; K13308 (desI), a component of four pathways linked to the biosynthesis of secondary metabolites; and K12645 (CYP170A), which belongs to two pathways linked to the biosynthesis of secondary metabolites.

## Discussion

The present study compared the effects of different bacterial consortia on the rhizosphere microbiome of soybean with those of a standard consortium commonly used in soybean production in South America. The results demonstrated that the rhizosphere selection of the microbiome is dependent on the composition of the applied consortium. It is well known that inoculation with single microbes can impact rhizosphere microbial community assembly, particularly the assembly of the bacterial community [55, 56]. However, our study is the first to show that inoculation with consortia of beneficial microbes with different plant growth-promoting traits can impact the assemblage of bacterial, archaeal and fungal communities directly affecting rhizofertility. The differences in rhizosphere microbial community assembly between the treatments are related to changes in rhizosoil fertility what might be linked to differences in root exudate production and release stimulated by the bacterial consortia. Root exudates provide a favorable environment for the growth of specific microbes, leading to increased abundance [57].

The program for selecting natural variants of *B. japonicum* and *B. diazoefficiens* adapted to tropical environmental conditions was detailed previously [58]. The strains used in this study are representative of the majority of inoculants applied to soybean crops in South America [9]. Inoculation with these strains is practiced even in areas that have received inoculants for several years, as annual inoculation increases the average grain yield by ~ 8%. *Azospirillum brasilense* strains Ab-V_5_ (= CNPSo 2083) and Ab-V_6_ (= CNPSo 2084) were selected, validated under field conditions and launched for use in microbial inoculants for co-inoculation of soybean in 2013 [8]. Nowadays, it is estimated that 25% of the area cultivated with soybean in Brazil (~ 10 million hectares) is co-inoculated with *Bradyrhizobium* and these two *A. brasilense* strains, which reduce the need for inorganic N fertilizers [9].

The mechanisms underlying the interactions of leguminous plants, particularly soybean, with soil microorganisms remain elusive. Comprehensive analyses of rhizosphere microbial community composition could support the optimal utilization of microbes in agriculture, particularly when combined with analyses of function. The rhizosphere effects may be influenced by the specific profile of root exudates, such as a high concentration of flavonoids, which are essential components of signal exchange between soybean and symbiotic rhizobia during nodule formation [59].

Although here we have not determined the profile of the root exudates, we have determined the rhizosphere soil fertility what was very different among the treatments. The sensitivity analysis and PCA performed in this study shed light on the impacts of the different bacterial consortia on soybean rhizosphere soil variables. Sensitivity analysis disentangles effects in a hierarchical manner [60]. The results of the sensitivity analysis showed that the effects of the different consortia of beneficial microbes were greatest for rhizosoil fertility, followed by the bacterial community, plant physiology, nutrition and yield, and the archaeal and fungal communities.

Rhizosoil fertility is influenced by plant metabolites and root exudates, which vary depending on the consortium used to inoculate soybean [56]. Several studies have shown that different plant species recruit specific sets of bacterial communities in the rhizosphere to optimize growth [61–63]. In the present study, *Bradyrhizobium* was abundant in the rhizosphere soil, which likely reflects the chemotaxis of these bacteria toward soybean root exudates. The rhizosphere soil was also highly abundant in potential PGPB genera such as *Bacillus*, which has been linked to systemic pathogen resistance, phosphorus solubilization, and antibiotic production [64].

At the genus level, *Bradyrhizobium* was consistently enriched in the soybean rhizosphere in the treatments with bacterial consortia, mainly in SI + Az, consistent with the notion that copiotrophs such as *Actinobacteria* and *Proteobacteria* were more competitive in a nutrient-enriched environment like the rhizosphere, whereas oligotrophs such as *Acidobacteria* and *Verrucomicrobia* are more abundant in soil with poor nutrient levels [65] and undisturbed forest soil [66]. *Bradyrhizobium*, *Bacillu*s and *Stenotrophomonas* were more abundant in the rhizosphere at the flowering stage of soybean than at the vegetative and mature stages [2]. In the present study, the abundance of functional genes related to BNF (*nifH*, *nifD*) was higher in rhizosphere soil than in bulk soil, consistent with previous findings [67], and increased further in the SI + Az treatment.

The treatments significantly impacted both high- and low-abundance taxa in soybean rhizosphere soil. Interestingly, each treatment exhibited a specific shifts in the numbers of high- and low-abundance functions. This indicates that different bacterial consortia may have unique effects on the microbial community and functional potential in rhizosphere soil. Previous studies have shown that microbial rarity is context-dependent, influenced by both biotic and abiotic factors such as soil inorganic nitrogen and microbial carbon use efficiency [68, 69]. Our current study expands on these findings by showing that inoculating different microbial consortia leads to shifts in the rhizosphere microbial community and its functional pool.

In terms of specific taxa and functions, SI + Az primarily positively modulated *Azospirillum*, *Burkholderia* and *Saccharibacteria* and several functions linked to secondary metabolites, amino acids, lipopolysaccharide biosynthesis, photosynthesis, bacterial secretion systems, and sulfur metabolism. By contrast, SI + Bs mainly affected *Nitrobacter* and *Glycomyces*, which are associated with the biosynthesis of secondary metabolites and amino acids. Furthermore, SI + MSM positively influenced *Pimelobacter*, *Terrabacter*, *Catenulispora*, and *Rhizobium*, which are associated with bacterial secretion systems, the biosynthesis of secondary metabolites, and amino acids. These findings suggest that different bacterial consortia have distinct effects on the rhizosphere microbiome of soybean plants, with potential implications for plant growth, nutrient uptake, and disease tolerance [70].

Chlorophyll concentration is an important physiological attribute that is directly related to the photosynthetic rate [21]. In this study, the concentrations of photosynthetic pigments (chlorophylls and carotenoids) differed significantly between treatments, the increase in the chl *a* content may be related to a greater supply of N by N-fixing microorganisms [71]. The chl *b* acts as an accessory pigment in photosynthesis and expands the light range used in this process. Increases in the concentration of chl *b* increase light absorption and the photosynthetic rate, thereby increasing productivity and grain quality [72]. In our study, SI + Az and SI + MSM significantly increased soybean leaf chlorophyll content, suggesting that this consortium can mitigate environmental stress and increase the activity of photosynthesis-related electron transporters.

Overall, our results indicate that SI + Az provided the best performance in terms of plant nutrition, physiology, yield and grain quality. The positive effects of *A. brasilense* co-inoculation on plant growth are best explained by the “multiple mechanisms theory” or additive hypothesis [73]. In other words, the plant growth-promoting effects of *Azospirillum* sp. are the result of a combination of mechanisms rather than a single mechanism [74]. Nevertheless, our data clearly indicate the importance of niche-based processes in structuring the rhizospheric community.

## Conclusions

Our study provides valuable insights into the dynamics of the soybean rhizosphere microbiome and its response to bacterial consortia. The different bacterial consortium treatments had significant effects on the soybean holobiont, particularly the rhizomicrobiome and rhizosoil fertility. The differential impacts of the treatments on the dependent variables demonstrate that selecting appropriate bacterial consortia for specific outcomes is crucial. This conclusion is consistent with niche-based theory on the role of selection by the plant and other environmental variables in the assemblage of the microbial community at both the taxonomic and functional levels. These findings have important implications for developing microbial-based agricultural practices that enhance crop productivity, crop quality and sustainability. However, further studies are needed to fully understand the underlying mechanisms and explore the potential of different bacterial consortia in various agricultural systems.

### Electronic supplementary material

Below is the link to the electronic supplementary material.

Supplementary Material 1

## Data Availability

The sequences were submitted to the European Nucleotide Archive (ENA) and are available under accession number PRJEB31659.
