# Supplementary material for: Diverse bacterial consortia: key drivers of rhizosoil fertility modulating microbiome functions, plant physiology, nutrition, and soybean grain yield
Source: Environ Microbiome. 2024 Jul 19;19:50. doi: 10.1186/s40793-024-00595-0 (PMC11264919; doi:10.1186/s40793-024-00595-0)
Supplement: Supplementary file 1 — Supplementary Material 1 [file 40793_2024_595_MOESM1_ESM.docx]

**Supplementary figure**


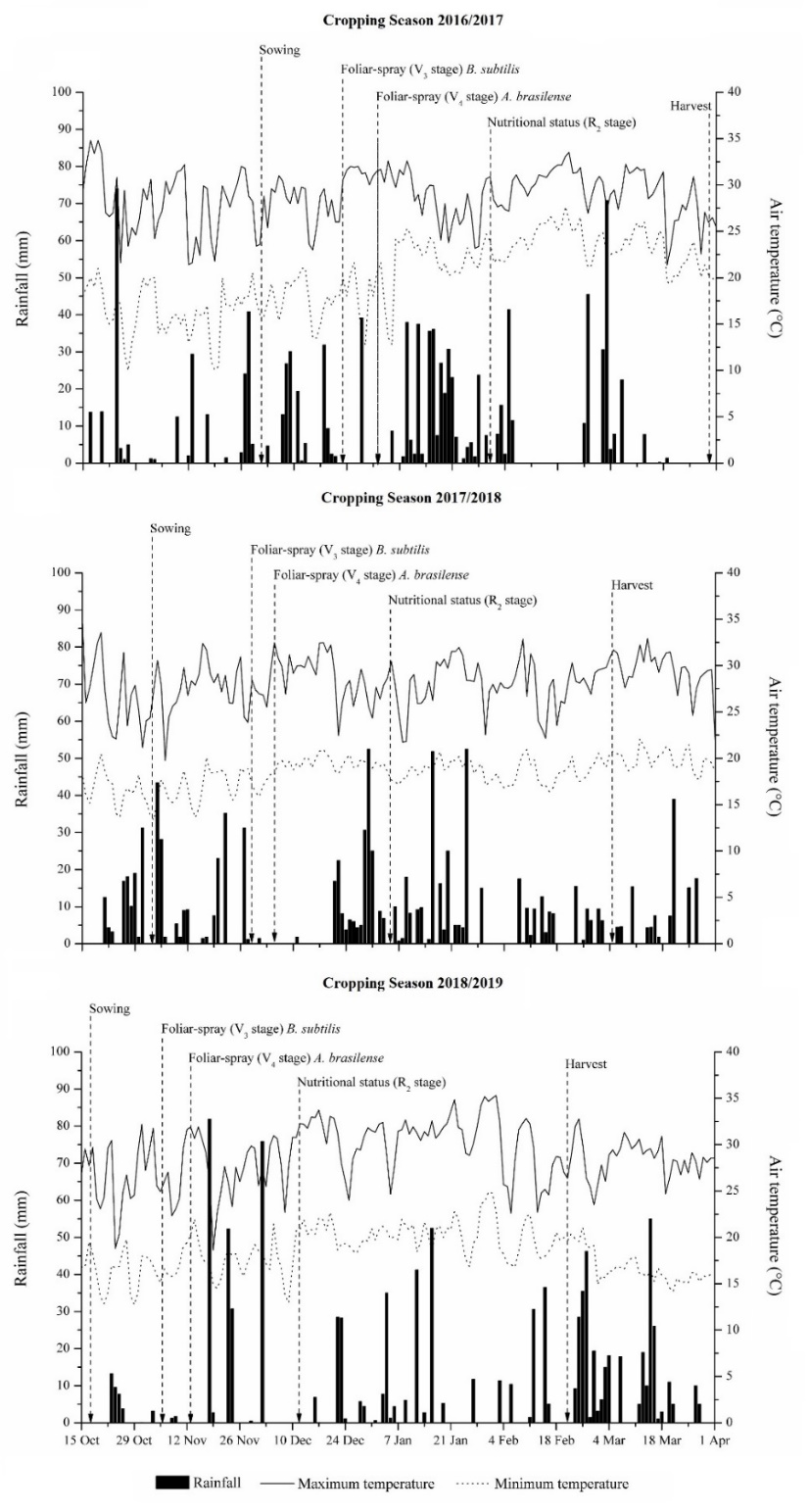


**Figure S1.** Rainfall and maximum and minimum air temperatures during the experimental period. Lageado Farm, Botucatu, São Paulo State, Brazil, 2016–2019.

# Supplementary tables

**Table S1.** Identification of samples in the European Nucleotide Archive and number of sequencing reads.

| **Treatment** | **Soil Type** | **Library** | **ENA ID** | | **Reads**^§^ |
| --- | --- | --- | --- | --- | --- |
| *i*^†^ | Bulk Soil | A | ERS3223779 | ERS3223780 | 9,096,933 |
|  |  |  | ERS3223781 | ERS3223782 |  |
|  |  | B | ERS3223783 | ERS3223784 | 12,029,397 |
|  |  |  | ERS3223785 | ERS3223786 |  |
|  |  | C | ERS3223787 | ERS3223788 | 12,008,695 |
|  |  |  | ERS3223789 | ERS3223790 |  |
| *ii* | Rhizosphere | A | ERS3223791 | ERS3223792 | 9,115,964 |
|  |  |  | ERS3223793 | ERS3223794 |  |
|  |  | B | ERS3223795 | ERS3223796 | 7,688,850 |
|  |  |  | ERS3223797 | ERS3223798 |  |
|  |  | C | ERS3223799 | ERS3223800 | 9,852,427 |
|  |  |  | ERS3223801 | ERS3223802 |  |
| *iii* | Rhizosphere | A | ERS3223803 | ERS3223804 | 6,748,449 |
|  |  |  | ERS3223805 | ERS3223806 |  |
|  |  | B | ERS3223807 | ERS3223808 | 7,754,378 |
|  |  |  | ERS3223809 | ERS3223810 |  |
|  |  | C | ERS3223811 | ERS3223812 | 5,585,161 |
|  |  |  | ERS3223813 | ERS3223814 |  |
| *iv* | Rhizosphere | A | ERS3223815 | ERS3223816 | 7,161,724 |
|  |  |  | ERS3223817 | ERS3223818 |  |
|  |  | B | ERS3223819 | ERS3223820 | 7,424,390 |
|  |  |  | ERS3223821 | ERS3223822 |  |
|  |  | C | ERS3223823 | ERS3223824 | 8,452,877 |
|  |  |  | ERS3223825 | ERS3223826 |  |
| *v* | Rhizosphere | A | ERS3223827 | ERS3223828 | 6,123,140 |
|  |  |  | ERS3223829 | ERS3223830 |  |
|  |  | B | ERS3223831 | ERS3223832 | 7,848,494 |
|  |  |  | ERS3223833 | ERS3223834 |  |
|  |  | C | ERS3223835 | ERS3223836 | 6,480,333 |
|  |  |  | ERS3223837 | ERS3223838 |  |

**^§^**Reads per library, four runs per library

**Table S2.** Soybean rhizosphere chemical attributes in the different inoculation treatments. Botucatu, São Paulo State, Brazil, 2019.

| **Attributes** | | **Rhizosphere^†^** | | | | ***P* > F** |
| --- | --- | --- | --- | --- | --- | --- |
|  |  | SI | SI + MSM | SI + Bs | SI + Az |  |
| pH | (CaCl_2_) | 5.2 a | 5.2 a | 5.2 a | 5.1 a | *^ns^* |
| P | (mg kg^-1^) | 32.5 b | 32.8 b | 33.0 b | 38.3 a | ** |
| K^+^ | (mmol_c_ kg^-1^) | 4.0 a | 4.2 a | 4.1 a | 4.1 a | *^ns^* |
| Ca^2+^ | (mmol_c_ kg^-1^) | 33.5 a | 34.2 a | 34.6 a | 34.0 a | *^ns^* |
| Mg^2+^ | (mmol_c_ kg^-1^) | 21.3 a | 22.5 a | 21.9 a | 22.3 a | *^ns^* |
| Al^3+^ | (mmol_c_ kg^-1^) | <1 | <1 | <1 | <1 | *^ns^* |
| H + Al | (mmol_c_ kg^-1^) | 32.4 a | 31.5 a | 33.2 a | 32.0 a | *^ns^* |
| S-SO_4_ ^2-^ | (mg kg^-1^) | 5.0 b | 5.1 b | 5.2 b | 5.6 a | ** |
| EB^a^ | (mmol_c_ kg^-1^) | 58.6 a | 60.7 a | 60.5 a | 60.5 a | *^ns^* |
| CEC^b^ | (%) | 91.0 a | 92.2 a | 93.5 a | 92.5 a | *^ns^* |
| BS^c^ | (mmol_c_ kg^-1^) | 64.5 a | 65.5 a | 64.6 a | 65.4 a | *^ns^* |
| B | (mg kg^-1^) | <1 | <1 | <1 | <1 | *^ns^* |
| Cu | (mg kg^-1^) | 9.5 b | 9.5 b | 10.0 b | 10.5 a | ** |
| Fe | (mg kg^-1^) | 24.3 b | 23.3 b | 25.3 b | 26.3 a | ** |
| Mn | (mg kg^-1^) | 56 b | 60 b | 60 b | 70 a | ** |
| Zn | (mg kg^-1^) | 2.4 b | 2.7 a | 2.9 a | 3.0 a | *^ns^* |
| SOM^d^ | (g kg^-1^) | 24.0 a | 23.8 a | 24.3 a | 24.2 a | *^ns^* |

^a^EB - Exchangeable base; ^b^BS - Base saturation; ^c^CEC - Cation exchange capacity; ^d^SOM - Soil organic matter.

**†**Inoculation treatments: SI = standard inoculation of seeds with *Bradyrhizobium japonicum* strain SEMIA 5079 and *B.* *diazoefficiens* strain SEMIA 5080; MSM = application of microbial secondary metabolites enriched in LCOs extracted from *B. diazoefficiens* strain USDA 110 and *Rhizobium tropici* strain CIAT 889 to seeds; *B. subtilis* = foliar spraying with *Bacillus subtilis* strain QST 713 at stage V_3_; *A. brasilense* = foliar spraying with *Azospirillum brasilense* strains Ab-V5 and Ab-V6 at stage V_4_. ‡ Values are means; *n* = 4. Means in the same row followed by different letters are significantly different by the LSD test. Significance levels: *ns*: *p* > 0.05; **p* < 0.05; ** *p* < 0.001.

**Table S3.** Nutrient and N-ureide (U) concentrations in soybean leaves with petioles in the different inoculation treatments during three cropping seasons. Botucatu, São Paulo State, Brazil, 2016/2019.

| **Factors** | **N** | | **P** | **K** | **Ca** | **Mg** | **S** | **Cu** | **Fe** | **Zn** | **Mn** | **B** | **U** |
| --- | --- | --- | --- | --- | --- | --- | --- | --- | --- | --- | --- | --- | --- |
| **Inoculation (I†)** | | ------------------------- g kg^-1^------------------------- | | | | | | ------------------- mg kg^-1^ ----------------- | | | | | µ mol g^-1^ |
| Standard Inoculation (SI) | | 42 b^‡^ | 2.8 b | 22 a | 10.0 a | 3.5 a | 2.7 a | 8.4 a | 125 d | 25 a | 51 c | 58 a | 10.2 c |
| SI + Microbial Secondary Metabolites | | 42 b | 3.0 b | 24 a | 10.2 a | 3.6 a | 2.8 a | 8.5 a | 136 b | 26 a | 56 b | 60 a | 10.7 b |
| SI + *B. subtilis* | | 42 b | 3.0 b | 23 a | 10.0 a | 3.6 a | 2.8 a | 8.5 a | 132 b | 26 a | 52 c | 59 a | 10.4 c |
| SI + *A. brasilense* | | 45 a | 3.2 a | 23 a | 10.1 a | 3.7 a | 2.8 a | 8.6 a | 141 a | 27 a | 60 a | 59 a | 11.9 a |
| **Cropping Season (CS)** | | | | | | | | | | | | | |
| 2016−2017 | | 43 a | 2.8 a | 24 a | 10.2 a | 3.7 a | 2.8 a | 8.5 a | 135 a | 26 a | 52 a | 58 a | 10.6 a |
| 2017−2018 | | 43 a | 3.0 a | 23 a | 10.0 a | 3.6 a | 2.7 a | 8.5 a | 134 a | 26 a | 53 a | 59 a | 10.6 a |
| 2018−2019 | | 43 a | 2.9 a | 24 a | 10.1a | 3.5 a | 2.8 a | 8.6 a | 133 a | 26 a | 52 a | 58 a | 10.4 a |
| **ANOVA *(F probability)*** | | | | | | | | | | | | | |
| I | | ** | * | *^ns^* | *^ns^* | *^ns^* | *^ns^* | *^ns^* | * | *^ns^* | * | * | ** |
| CS | | *^ns^* | *^ns^* | *^ns^* | *^ns^* | *^ns^* | *^ns^* | *^ns^* | *^ns^* | *^ns^* | *^ns^* | *^ns^* | *^ns^* |
| I*CS | | *^ns^* | *^ns^* | *^ns^* | *^ns^* | *^ns^* | *^ns^* | *^ns^* | *^ns^* | *^ns^* | *^ns^* | *^ns^* | *^ns^* |

**†**Inoculation treatments: SI = standard inoculation of seeds with *Bradyrhizobium japonicum* strain SEMIA 5079 and *B.* *diazoefficiens* strain SEMIA 5080; MSM = application of microbial secondary metabolites enriched in LCOs extracted from *B. diazoefficiens* strain USDA 110 and *Rhizobium tropici* strain CIAT 889 to seeds; *B. subtilis* = foliar spraying with *Bacillus subtilis* strain QST 713 at stage V_3_; *A. brasilense* = foliar spraying with *Azospirillum brasilense* strains Ab-V5 and Ab-V6 at stage V_4_. ‡ Values are means; *n* = 4. Means in the same column followed by different letters are significantly different by the LSD test. Significance levels: *ns*: *p* > 0.05; **p* < 0.05; ** *p* < 0.001.

**Table S4.** Chlorophyll *a* (Chl *a*), chlorophyll *b* (Chl *b*), total chlorophylls (Total Chl), and total carotenoids (Total Car) in soybean leaves (R_2_ phenological stage) in the different inoculation treatments during three cropping seasons. Botucatu, São Paulo State, Brazil, 2016/2019.

| **Factors** | **Chl *a*** | **Chl *b*** | **Total Chl** | **Total Car** |
| --- | --- | --- | --- | --- |
| **Inoculation (I†)** | μg·cm^−2^ |  |  |  |
| Standard Inoculation (SI) | 15.0 b^‡^ | 3.0 b | 18.0 b | 4.6 a |
| SI + Microbial Secondary Metabolites | 16.5 a | 3.9 a | 20.4 a | 4.6 a |
| SI + *B. subtilis* | 15.1 b | 3.0 b | 18.1 b | 5.7 a |
| SI + *A. brasilense* | 16.9 a | 3.8 a | 20.7 a | 5.6 a |
| **Cropping Season (CS)** |  |  |  |  |
| 2016−2017 | 15.5 b | 3.4 a | 18.9 b | 5.0 a |
| 2017−2018 | 16.1 a | 3.5 a | 20.6 a | 5.3 a |
| 2018−2019 | 15.8 b | 3.5 a | 19.2 b | 5.1 a |
| **ANOVA *(F probability)*** |  |  |  |  |
| I | ** | ** | ** | *^ns^* |
| CS | * | *^ns^* | * | *^ns^* |
| I*CS | *^ns^* | *^ns^* | *^ns^* | *^ns^* |

**†**Inoculation treatments: SI = standard inoculation of seeds with *Bradyrhizobium japonicum* strain SEMIA 5079 and *B.* *diazoefficiens* strain SEMIA 5080; MSM = application of microbial secondary metabolites enriched in LCOs extracted from *B. diazoefficiens* strain USDA 110 and *Rhizobium tropici* strain CIAT 889 to seeds; *B. subtilis* = foliar spraying with *Bacillus subtilis* strain QST 713 at stage V_3_; *A. brasilense* = foliar spraying with *Azospirillum brasilense* strains Ab-V5 and Ab-V6 at stage V_4_. ‡ Values are means; *n* = 4. Means in the same column followed by different letters are significantly different by the LSD test. Significance levels: *ns*: *p* > 0.05; **p* < 0.05; ***p* < 0.001.

**Table S5.** Net photosynthetic rate (*A*), stomatal conductance (*g_S_*), internal CO_2_ concentration in the substomatal chamber (*Ci*), transpiration (*E*), water use efficiency (WUE), and carboxylation efficiency (Cef) of soybean leaves (R_2_ phenological stage) in the different inoculation treatments during three cropping seasons. Botucatu, São Paulo State, Brazil, 2016/2019.

| **Factors** | ***A*** | ***gs*** | ***Ci*** | | ***E*** | | **WUE** | **Cef** |
| --- | --- | --- | --- | --- | --- | --- | --- | --- |
| **Inoculation (I†)** | μmol CO_2_ m^−2^ s^−1^ | mol H_2_O m^−2^ s^−1^ | μmol mol^−1^ | | μmol CO_2_ (mmol H_2_O) ^−1^ | | μmol CO_2_ (mmol H_2_O)^−1^) | *A*/*Ci* |
| Standard Inoculation (SI) | 25.3 b^‡^ | 0.177 b | 254 b | | 2.5 a | | 10.1 b | 0.099 a |
| SI + Microbial Secondary Metabolites | 28.5 a | 0.173 b | 255 b | | 2.5 a | | 11.4 a | 0.112 a |
| SI + *B. subtilis* | 25.8 b | 0.196 b | 263 b | | 2.6 a | | 9.9 b | 0.098 a |
| SI + *A. brasilense* | 29.2 a | 0.270 a | 270 a | | 2.5 a | | 11.6 a | 0.108 a |
| **Cropping Season (CS)** |  |  |  | |  | |  |  |
| 2016−2017 | 26.9 b | 0.223 b | 257 a | | 3.0 a | | 9.8 b | 0.099 b |
| 2017−2018 | 27.5 a | 0.230 a | 265 a | | 3.1 a | | 10.4 b | 0.110 b |
| 2018−2019 | 27.6 a | 0.221 b | 260 a | | 3.1 a | | 10.9 a | 0.116 a |
| **ANOVA *(F probability)*** |  |  | |  | |  |  |  |
| I | * | * | * | | *^ns^* | | ** | *^ns^* |
| CS | * | * | *^ns^* | | *^ns^* | | * | * |
| I*CS | *^ns^* | * | *^ns^* | | *^ns^* | | *^ns^* | *^ns^* |

**†**Inoculation treatments: SI = standard inoculation of seeds with *Bradyrhizobium japonicum* strain SEMIA 5079 and *B.* *diazoefficiens* strain SEMIA 5080; MSM = application of microbial secondary metabolites enriched in LCOs extracted from *B. diazoefficiens* strain USDA 110 and *Rhizobium tropici* strain CIAT 889 to seeds; *B. subtilis* = foliar spraying with *Bacillus subtilis* strain QST 713 at stage V_3_; *A. brasilense* = foliar spraying with *Azospirillum brasilense* strains Ab-V5 and Ab-V6 at stage V_4_. ‡ Values are means; *n* = 4. Means in the same column followed by different letters are significantly different by the LSD test. Significance levels: *ns*: *p* > 0.05; **p* < 0.05; ***p* < 0.001.

**Table S6.** Nodule number (NN), nodule dry weight (NDW), root dry weight (RDW), shoot dry weight (SDW), 100-grain weight (100-GW), grain yield (GY), crude protein (CP), and agronomic efficiency index (AEI) of soybean in the different inoculation treatments during three cropping seasons. Botucatu, São Paulo State, Brazil, 2016/2019.

| **Factors** | **NN** | **NDW** | **RDW** | **SDW** | **100-GW** | **GY** | **CP** | **AEI**§ |
| --- | --- | --- | --- | --- | --- | --- | --- | --- |
| **Inoculation (I†)** | *n° plant^-1^* | *mg plant^-1^* | *-----------g plant^-1^----------* | | *g* | *kg ha^-1^* | *g kg^-1^* | % |
| Standard Inoculation (SI) | 50 b^‡^ | 210 b | 5.2 b | 18.2 b | 16.4 b | 4780 b | 422 a | - |
| SI + Microbial Secondary Metabolites | 55 b | 220 b | 5.3 b | 18.3 b | 16.5 b | 4895 b | 426 a | + 2.4 |
| SI + *B. subtilis* | 52 b | 214 b | 5.2 b | 18.4 b | 16.4 b | 4851 b | 422 a | + 1.5 |
| SI + *A. brasilense* | 60 a | 241 a | 5.7 a | 18.9 a | 16.7 a | 5086 a | 429 a | + 6.4 |
| **Cropping Season (CS)** | | | | | | | | |
| 2016−2017 | 53 b | 223 b | 5.3 a | 18.4 b | 16.3 b | 4790 b | 42.6 a | - |
| 2017−2018 | 58 a | 240 a | 5.4 a | 18.7 a | 16.9 a | 5241 a | 42.7 a | - |
| 2018−2019 | 55 b | 229 b | 5.3 a | 18.5 b | 16.5 b | 4863 b | 42.5 a | - |
| **ANOVA *(F probability)*** | | | | | | | | |
| I | ** | ** | ** | * | * | ** | *^ns^* | - |
| CS | * | * | *^ns^* | * | * | * | *^ns^* | - |
| I*CS | *^ns^* | *^ns^* | *^ns^* | *^ns^* | *^ns^* | *^ns^* | *^ns^* | - |

**†**Inoculation treatments: SI = standard inoculation of seeds with *Bradyrhizobium japonicum* strain SEMIA 5079 and *B.* *diazoefficiens* strain SEMIA 5080; MSM = application of microbial secondary metabolites enriched in LCOs extracted from *B. diazoefficiens* strain USDA 110 and *Rhizobium tropici* strain CIAT 889 to seeds; *B. subtilis* = foliar spraying with *Bacillus subtilis* strain QST 713 at stage V_3_; *A. brasilense* = foliar spraying with *Azospirillum brasilense* strains Ab-V5 and Ab-V6 at stage V_4_. ‡ Values are means; *n* = 4. Means in the same column followed by different letters are significantly different by the LSD test. Significance levels: *ns*: *p* > 0.05; **p* < 0.05; ***p* < 0.001. §AEI = Agronomic efficiency index of the bacterial consortium relative to the standard inoculation.
